# Quality of life of Australian chronically-ill adults: patient and practice characteristics matter

**DOI:** 10.1186/1477-7525-7-50

**Published:** 2009-06-03

**Authors:** Upali W Jayasinghe, Judith Proudfoot, Christopher A Barton, Cheryl Amoroso, Chris Holton, Gawaine Powell Davies, Justin Beilby, Mark F Harris

**Affiliations:** 1 Centre for Primary Health Care and Equity, School of Public Health & Community Medicine, University of New South Wales, Sydney, New South Wales, Australia; 2 Discipline of General Practice, University of Adelaide, Adelaide, South Australia, Australia; 3 Faculty of Health Sciences, University of Adelaide, Adelaide, South Australia, Australia

## Abstract

**Background:**

To study health-related quality of life (HRQOL) in a large sample of Australian chronically-ill patients and investigate the impact of characteristics of patients and their general practices on their HRQOL and to assess the construct validity of SF-12 in Australia.

**Methods:**

Cross sectional study with 96 general practices and 7606 chronically-ill patients aged 18 years or more using standard SF-12 version 2. Factor analysis was used to confirm the hypothesized component structure of the SF-12 items. SF-12 physical component score (PCS-12) and mental component score (MCS-12) were derived using the standard US algorithm. Multilevel regression analysis (patients at level 1 and practices at level 2) was applied to relate PCS-12 and MCS-12 to patient and practice characteristics.

**Results:**

There were significant associations between lower PCS-12 or MCS-12 score and poorer general health (10.8 (regression coefficient) lower for PCS-12 and 7.3 lower for MCS-12), low socio-economic status (5.1 lower PCS-12 and 2.9 lower MCS-12 for unemployed, 0.8 lower PCS-12 and 1.7 lower MCS-12 for non-owner-occupiers, 1.0 lower PCS-12 for less well-educated) and having two or more chronic conditions (up to 2.7 lower PCS-12 and up to 1.5 lower MCS-12 than those having a single disease). Younger age was associated with lower MCS-12 (2.2 and 6.0 lower than middle age and older age respectively) but higher PCS-12 (4.7 and 7.6 higher than middle age and older age respectively). Satisfaction with quality of care (regression coefficient = 1.2) and patients who were married or cohabiting (regression coefficient = 0.6) was positively associated with MCS-12. Patients born in non-English-speaking countries were more likely to have a lower MCS-12 (1.5 lower) than those born in Australia. Employment had a stronger association with the quality of life of males than that of females. Those attending smaller practices had lower PCS-12 (1.0 lower) and MCS-12 (0.6 lower) than those attending larger practices. At the patient level (level 1) 42% and 21% of the variance respectively for PCS-12 and MCS-12 were explained by the patients and practice characteristics. At the practice level (level 2), 73% and 49% of the variance respectively for PCS-12 and MCS-12 were explained by patients and practice characteristics.

**Conclusion:**

The strong association between patient characteristics such as socio-economic status, age, and ethnicity and SF-12 physical and mental component summary scores underlines the importance of considering these factors in the management of chronically-ill patients in general practice. The SF-12 appears to be a valid measure for assessing HRQOL of Australian chronically-ill patients.

## Background

In 2004, 77% of Australians reported having at least one long term medical condition [1]. Patients with chronic conditions account for an increasing burden of disease and presentations in general practice in Australia [2,3] and the proportion of encounters for both diabetes and cardiovascular disorders is increasing [3]. The management of chronic illness has thus become a major focus in general practice, both because of its prevalence and the opportunity which general practice has to intervene early to improve quality of life, prevent disability and reduce hospital use. Since 1999, the Australian government has introduced a variety of strategies to improve the care of people with chronic illness [4]. Having effective ways of assessing the health status of patients is critical to the evaluation and monitoring of these strategies [5].

The measurement of health-related quality of life (HRQOL) from the perspective of the patient has become a major aspect of health services evaluation [6]. The standardized measurement of health outcomes, through instruments such as the SF-36, and more recently the SF-12, has had significant benefit for all fields and professions concerned with health [7,8]. In particular, standardized assessment of health status is valuable for assessing the effectiveness of medical interventions, for monitoring the progress of patients in clinical settings, and for evaluating health and well-being at the population level [9]. Investigators from numerous countries representing diverse cultures have determined that the SF-36 and SF-12 are sensitive to differences in a number of socio-demographic and clinical variables, including age [8,10], gender [8,11], income [7,11-13], employment [7,11,14], education [7,9,12,14], self reported general health [10], marital status [15], ethnicity [6,9] and number of conditions [10,12].

The study aimed to examine variations in the two subscales of the SF-12 ('physical component score (PCS-12)' and 'mental component score (MCS-12)') according to practice and patient characteristics as well as satisfaction with care and the number of medical conditions in a population of chronically-ill patients attending Australian general practice. It also examined the construct validity of SF-12 in this population.

## Methods

### Participants

This study was part of a larger study examining the impact of the organizational capacity of general practices in Australia to manage chronic diseases. It was conducted in 27 Divisions (local primary care support organizations) in five states and in the Australian Capital Territory between December 2003 and October 2004. The data on Division characteristics showed that the 27 were representative of the 103 Divisions approached except that recruited general practices from 27 Divisions tended to be larger and to have a lower population to general practitioners ratio than the Australian average [16]. In each practice, clinical management software was used to select a random sample of 180 patients aged 18 years or more currently being prescribed medication for three common chronic diseases: asthma, type 2 diabetes, and hypertension/ischaemic heart disease. Practices were permitted to remove patients from the list who were deceased or otherwise inappropriate to invite. A total of 12,544 patients attending 96 practices were invited to participate. Completed surveys were received from 7606 patients (a response rate of 61%). A priori sample size calculations on the SF-12 physical component score confirmed that after adjustment for clustering (previous studies on SF-36 indicated a cluster effect (ICC = Intra-cluster correlation) of 0.011 for the PCS-36 [14]) predicted that an average of 50 patients from each of 100 practices would have sufficient power (1-β = 0.8 and α = 0.05) to detect an effect size of 0.10 between patients with good and poor general health assuming that about half of the patients were in good general health.

### Ethics

Ethics approval for the study was obtained from the University of New South Wales (UNSW) Human Research Ethics Committee and University of Adelaide Human Research Ethics Committee. Both practice staff and patients provided written informed consent.

### Instruments

The standard SF-12 version 2 is a 12-item questionnaire measuring physical and mental health [6,12]. The adoption of the SF-12 version 2 over the original version 1 form for all new studies including population surveys is recommended [17]. It is an abbreviated form of the SF-36 Health Survey, which is one of the most widely used instruments for assessing HRQOL [12]. Both instruments produce eight dimensions of health (physical functioning (PF), role physical (RF), bodily pain (BP), general health (GH), vitality (VT), social functioning (SF), role emotional (RE), and mental health (MH)) [18,19]. They also produce two summary scores – the Physical Component Summary (PCS) and the Mental Health Component Summary (MCS) – and have been validated for use in the USA, UK and many other European countries for large scale health measurement and monitoring [12,19]. For ease of interpretation, scores are standardized to population norms, with the mean score set at 50 (SD = 10): higher scores indicate better health. The SF-12 has been shown to have good validity and reliability [17]. Previous research has supported the use of the standard SF-12 in Australian settings, rather than development of an 'Australian' short-form [20,21]. The SF-12 is an instrument that can be administered in three minutes with a small trade off between brevity and precision [21].

The same sample of patients completed the General Practice Assessment Survey (GPAS) version 2 [22] along with the SF-12. The patient characteristics including self-reported general health and chronic medical condition/conditions were collected using the GPAS. Patient satisfaction was also assessed through the GPAS. The GPAS is a multi-item self-report questionnaire which measures several dimensions relating to patients' assessment of general practice. The psychometric properties of the GPAS have been evaluated [23].

### Data and variables

The dependent variables were PCS-12 and MCS-12. Because patients do not register with general practitioners (GPs) in Australia, it was not possible to determine the "list size" of practices accurately and thus the number of general practitioners in a practice was used as a measure of the practice size. Geographical area was defined by using the Rural, Remote and Metropolitan Area (RRMA) classification [24] as urban (all metropolitan centers with populations ≥ 100,000) or rural (rural centers and all other areas with populations of less than 100,000). There were no practices in the sample which were zoned as remote. The socio-demographic characteristics of respondents studied were gender, age, self-reported general health status in the last 12 months, home ownership, education, employment, marital status, country of birth, disease and overall satisfaction with care (Table 1). Home ownership can be considered as one marker of economic status [25]. For some respondents, their specific chronic disease or diseases were not known and therefore 'unknowns' were included in the analysis as a separate category to minimize the data loss.

**Table 1:** Unadjusted mean and standard deviation of PCS-12 and MCS-12 scores by characteristics of practices and patients (number of patients = 7606; number of practices = 96)

Variable (definition)	Responses		PCS-12		MCS-12	
	No.	%	Mean (SD)	P-value	Mean (SD)	P-value
Characteristics of practices						
1–3 general practitioners	3970	52.2	41.8 (11.7)	< 0.001	48.8 (11.4)	0.016
4 or more general practitioners	3636	47.8	43.0 (11.9)		49.4 (10.8)	
Location of practice:						
Urban	4468	60.1	42.8 (11.8)	0.001	49.0 (11.0)	0.698
Rural	3038	39.9	41.8 (11.9)		49.2 (11.2)	
*Characteristics of patients*						
Gender:						
Male	3474	46.8	42.7(11.6)	0.16	49.8(10.9)	< 0.001
Female	3944	53.2	42.3(12.0)		48.5(11.3)	
Age (years):						
18–39 Yrs	749	10.1	50.4(9.3)	< 0.001	45.0(11.7)	< 0.001
40–59 Yrs	2538	34.3	44.7(11.4)		47.3(11.3)	
>59 yrs	4115	55.6	39.5(11.5)		51.0(10.5)	
Health status						
Good	4027	54.4	48.2 (8.9)	< 0.001	52.8 (8.9)	< 0.001
Poor	3382	45.6	35.5 (11.2)		44.6 (11.9)	
Home ownership						
Owner-occupied	5899	79.8	43.0 (11.7)	< 0.001	49.9 (10.7)	< 0.001
Rented	1496	20.2	40.4 (12.2)		46.0 (12.1)	
Education						
Degree/Diploma	2208	30.1	45.8 (11.2)	< 0.001	48.7 (11.1)	0.071
Elementary/High School	5138	69.9	41.0 (11.8)		49.3 (11.1)	
Employment						
Employed	2536	34.3	48.4 (9.2)	< 0.001	49.3 (10.2)	< 0.001
Retired	2935	39.7	39.9 (11.3)		51.4 (10.3)	
Unemployed (looking for work/full-time education/looking after family/unable to work due to sickness or disability)	1923	26.0	38.3 (12.5)		45.3 (12.4)	
Marital status						
Married (married/cohabiting)	5206	70.3	43.1 (11.6)	< 0.001	49.7 (10.8)	< 0.001
Unmarried (single/separated/divorced/widowed)	2200	29.7	41.0 (12.3)		47.6 (11.8)	
Country of birth						
Born in Australia	5474	74.6	42.6 (11.8)	0.008	49.2 (11.0)	0.001
Born in USA/UK/Canada/New Zealand	1001	13.7	42.8 (12.0)		49.6 (11.2)	
Born in non-English-speaking countries	858	11.7	41.3 (11.4)		47.8 (11.7)	
Disease						
Diabetes	1043	13.7	42.7 (10.9)	< 0.001	50.2 (10.4)	< 0.001
Ischaemic heart disease/hypertension	1404	18.5	40.5 (11.5)		49.3 (10.7)	
Asthma	792	10.4	42.8 (11.7)		47.7 (11.0)	
Two or more conditions	1497	19.7	36.1 (11.3)		47.3 (12.2)	
Disease unknown	2870	37.7	46.4 (11.0)		49.9 (10.8)	
Overall satisfaction with care						
High	2713	36.6	41.6 (12.3)	< 0.001	50.4 (11.3)	< 0.001
Low	4701	63.4	42.9 (11.5)		48.3 (11.0).	

Notes:

Unknowns were: Gender = 188, Age = 204, Health Status = 197, Home ownership = 211, Education = 260, Employment = 212, Marital status = 200, Country of birth = 273 and overall satisfaction = 192.

P-values are for comparison of component scores for categories of each characteristics using analysis of variance.

Patient characteristics were collected independently using GPAS^22 ^for the same respondents.

### Statistical analyses

Summary physical (PCS-12) and mental (MCS-12) components were constructed using the standard SF-12 version 2 US algorithm empirically derived from the data of a US general population survey [17]. To confirm the dimensions as documented by Kontodimopoulos et al. [26] and Ware et al. [17], we carried out a factor analysis using SPSS statistical software (version 15; SPSS, Chicago, IL, USA) with principal components analysis using the varimax rotation [26]. The number of factors was determined by the scree test and eigen values > 1. The two principal components were then rotated into simple orthogonal structures, a procedure previously implemented in similar studies [26]. It was hypothesized that two factors would be obtained (Table 2) known as physical health and mental health. In addition, items originally belonging to the PF, RP, BP and GH domains were hypothesized to load (or correlate) higher on the physical health factor, whereas the MH, RE, SF and VT items were hypothesized to relate most strongly to the mental health factor. However, VT and SF have been shown to load on both physical and mental components [26].

**Table 2:** Scores and rotated factor loadings for items SF-12 scales

Scale	Desciption of items	Lowest possible score % (No.)	Highest possible score % (No.)	Mean (SD)	Factor structure	
					Factor 1	Factor 2
PF*	Health limited in moderate activities	17.1 (1294)	48.0 (3638)	2.31 (0.74)	**0.85**	0.14
PF*	Health limit climbing stairs	25.5 (1916)	35.8 (2689)	2.10 (0.78)	**0.82**	0.09
RP	Accomplished less because of physical health	6.9 (525)	31.0 (2352)	3.57 (1.25)	**0.84**	0.30
RP	Limited in the kind of work	7.3 (551)	33.3 (2517)	3.62 (1.26)	**0.87**	0.27
BP	Pain interfered with activities	3.7(279)	35.6(2701)	3.74 (1.21)	**0.71**	0.30
GH	Health in general	5.4(411)	3.0(231)	3.13 (1.04)	**0.67**	0.33
MH	Felt calm and peaceful	3.5 (262)	10.5 (793)	3.50 (0.95)	0.10	**0.78**
MH	Felt downhearted and depressed	2.0 (151)	36.7 (2782)	3.93 (1.03)	0.07	**0.83**
RE	Accomplished less due to emotional problems	3.7 (277)	45.5 (3450)	3.97 (1.15)	0.39	**0.78**
RE	Did work less careful than usual due to emotional problems	3.0 (228)	48.8 (3691)	4.07 (1.10)	0.40	**0.73**
SF	Emotional problems/physical health interfered with social activities	3.0(229)	50.0 (3797)	4.04 (1.14)	0.46	**0.68**
VT	Lot of energy	9.4 (711)	4.2 (317)	3.00 (1.03)	**0.55**	0.49

Extraction method: Principal Component Analysis. Rotation method: Varimax with Kaiser Normalization. Rotation converged in 3 iterations.

*Two items of PF are three point scale (from 1 to 3) and all the other items are five point scale (from 1 to 5).

Figures for 12 items do not include missing values which range from 1.1% to 1.6% except for the question about "climbing stars" with 2.1% missing values.

Higher loadings of each item on a factor are indicated in bold text.

First, we examined the association between the independent variables and physical or mental health component scores in univariate analyses with analysis of variance using SPSS (Table 1). The analysis of variance was conducted to compare unadjusted scores. The Pearson χ^2 ^– test was used to compare proportions analyzed and missing.

### Multilevel Models

Multilevel regression models were used with two dimensions (physical and mental component scores) as continuous dependent variables and general practice and patient characteristics, including the hypothesized interaction between gender and employment (based on the previous studies [15,27,28]), as the independent variables. Multilevel analysis (with MLwiN Software [29]) adjusted for clustering of patients (level 1) within practices (level 2) [11,14,30]. Initially, we fitted a baseline variance component model (no independent variables) for each of the response variables followed by the main model. The main model expands the baseline model by including patient and practice characteristics with the hypothesized interaction [15,27,28] as fixed effects. The interaction effect of independent variables was included in the model if their regression coefficients were significant (Table 3) and they showed a significant improvement to the model without the interaction.

**Table 3:** Estimates of regression coefficient of multilevel regression analysis for practice and patient characteristics (number of patients = 6997; number of practices = 96)

Parameters (reference category)	Estimate for the main model	
	Physical components score (PCS-12)	Mental components score (MCS-12)
	Regression Coefficients (Standard Error)	Regression Coefficients (Standard Error)
*Patient main effect*		
Intercept	35.44	33.74
Female patients (male)	2.78 (0.46)^‡^	1.54 (0.51)^†^
Age, years		
40–59 (18–39)	-4.65 (0.40)^‡^	2.22 (0.44)^‡^
>59 (18–39)	-7.64 (0.45)^‡^	6.02 (0.50)^‡^
Good or very good health (very bad, bad or fair health)	10.83 (0.23)^‡^	7.34 (0.25)^‡^
Owner-occupier (rented)	0.79 (0.29)^†^	1.72 (0.32)^‡^
College/university (elementary/high School)	1.02 (0.25)^‡^	-0.05 (0.28)
Employed patients (unemployed)	7.29 (0.48)^‡^	4.81 (0.52)^‡^
Retired patients (unemployed)	3.99 (0.48)^‡^	3.69 (0.53)^‡^
Married/cohabiting (single/separated/divorced/widowed)	0.47 (0.25)	0.63 (0.27)*
Born in Australia (non-English countries)	-0.68 (0.35)	1.46 (0.39)^‡^
Born in USA/UK/Canada/NZ (non-English countries)	0.23 (0.44)	0.87 (0.48)
Diabetes (two or more conditions)	2.66 (0.37)^‡^	1.49 (0.41)^‡^
Ischaemic heart disease/hypertension (two or more conditions)	1.24 (0.34)^‡^	0.55 (0.37)
Asthma (two or more conditions)	1.01 (0.42)*	1.42 (0.46)^†^
Disease unknown (two or more conditions)	3.83 (0.31)^‡^	1.22 (0.34)^‡^
Overall satisfaction with care	-0.30 (0.23)	1.20 (0.25)^‡^
Patient interaction effect		
Female × employed	-3.46 (0.59)^‡^	-3.34 (0.65)^‡^
Female × retired	-3.34 (0.58)^‡^	-1.38 (0.64)*
*Practice main effect*		
Size 1–3 general practitioners (4 or more GPs)	-0. 99 (0.27)^‡^	-0.55 (0.26)*
Urban (Rural)	0.53 (0.28)	0.16 (0.27)

Note: *P < 0.05, ^† ^P < 0.01, ^‡ ^P < 0.001

Interactions not shown in the table were not included in the model.

NZ = New Zealand

Patient characteristics were collected independently using GPAS^22 ^for the same respondents.

### Significance of parameters

Parameter estimates were tested by the t value, determined by dividing the estimated coefficients by their standard errors (Table 3) [29]. Because the two models were nested, we used -2 log likelihood, known as the "change in the deviance", which has a chi-square distribution to test whether the difference between the two models was statistically significant (Table 4).

**Table 4:** Estimated variances (and standard errors), percent explained variance and intra-cluster correlations for physical and mental component scores (number of patients = 6997; number of practices = 96)

Random parameters	Estimated variance		
	Baseline model	Full model	% Explained variance
*Physical component scores*			
			
Level 2, Practice variance	4.40 (0.92)^‡^	0.60 (0.25)*	72.9
Level 1, Patient variance	135.67 (2.31)^‡^	80.07 (1.36)^‡^	42.4
Intracluster correlation	0.031	0.007	
Deviance	54326.89	50565.05	
			
*Mental component scores*			
			
Level 2, Practice variance	1.32 (0.43)^†^	0.19 (0.21)	49.2
Level 1, Patient variance	121.59 (2.07)^‡^	97.0 (1.65)^‡^	20.9
Intracluster correlation	0.011	0.002	
Deviance	53501.64	51877.94	

Note: *P < 0.05, ^† ^P < 0.01, ^‡ ^P < 0.001

### Variance explained at each level

The baseline variance component model explained how the total variance was partitioned into variance between patients and practices (Table 4). The variance explained was estimated using the baseline model and main model [31]. Differences in the modeled variance indicate how much better a model can account for the variance at a specific level [32]. The formulas to calculate the proportion of variance explained are given by Snijders and Bosker [31] and Sixma et al. [32].

## Results

There were 7606 of 12544 patient questionnaires returned (61% return rate). We conducted analyses comparing proportions of respondents with non-respondents for gender and age (available for 90% and 84% of non-respondents respectively). The gender of respondents (53.3% were females) and non-respondents (53.6% were females) were similar (P = 0.76). Twenty percent of non-respondents were younger than 40 years, compared to 10% of respondents and 14% of the total sample (P < 0.001). The mean age of respondents and non-respondents was 59.1 (SD = 15.0) and 55.3 years (SD = 17.8) respectively. Data completeness was excellent for all SF-12 items, with less than 1.6% of respondents not responding to each question apart from the question about "climbing stars" which 2.1% did not complete.

### Factor analysis

Factor analysis suggested a two-factor solution (Table 2). These two factors account for approximately 68.1% of the variance in the twelve items of the SF-12.

Correlations between physical and mental summary scores were very low with 0.054 (principal components analysis with the varimax rotation gives uncorrelated factors). The overall mean of PCS-12 and MCS-12 of these chronically-ill respondents were 42.4 (SD = 11.8) and 49.1 (SD = 11.1) respectively.

Table 1 shows the characteristics of respondents and practices (independent variables). Almost one-half of the respondents were patients from large practices and 40% of respondents were from rural areas. The mean age was 60 years (range 18–96). The majority (53%) was female and nearly 80% owned their own homes. Only 34% of respondents were employed and 40% were retired. Seventy-four per cent were born in Australia, 14% in USA, UK, Canada or New Zealand and the remaining 12% in non-English-speaking countries.

The multilevel regression included only data from the questionnaires for which information on all relevant variables was available, resulting in a final sample size of 6997 (92%) patients from 96 practices. Pearson Chi-Squared tests indicated that proportions of practice size, practice location, gender and country of birth were similar between the records used in multilevel analyses and missing data (data not shown). There were small but significant differences between the proportions of records analyzed and the total (including missing) for other characteristics: 0.7% (age), 0.5% (general health status), 0.3% (home ownership), 0.4% (education), 0.7% (employment), 0.3% (marital status) and 0.4% (disease).

Table 3 shows the results of the multilevel regression analyses for each of the response variables.

Patient characteristics including self-rated general health and chronic medical conditions were collected independently using GPAS^22 ^for the same respondents (Table 1). Patients' assessment of overall satisfaction with care was also assessed through the GPAS. PCS-12 declined with age, but in contrast MCS-12 increased with age. Patients with better self-reported general health status rated both PCS-12 and MCS-12 higher than those with poor general health (Table 3). Both self-reported PCS-12 and MCS-12 were positively related to home ownership. Well-educated patients tended to rate PCS-12 higher than less well-educated patients, but there was no association with MCS-12.

**Figure 1:**
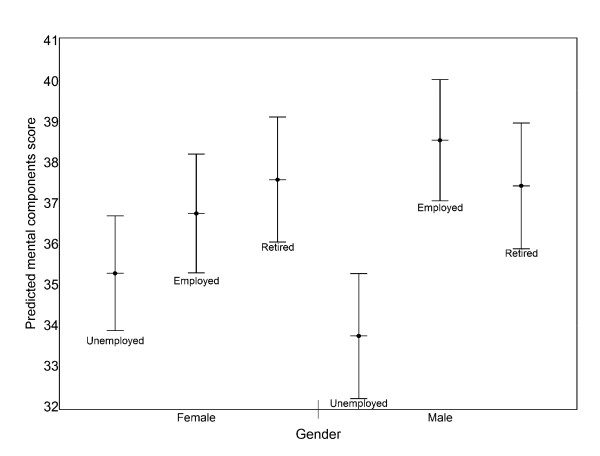
**95% confidence intervals for predicted MCS-12 (Mean ± 1.96 SE) by gender and employment status**. Predicted values were based on the multilevel regression model with interaction between gender and employment status.

Patients who were employed or retired were likely to have higher PCS-12 and MCS-12 than unemployed. Gender interacted with employment in predicting both PCS-12 and MCS-12 with unemployment being more associated with poorer health in males than in females (Figure 1).

Patients who were married or cohabiting tended to have higher MCS-12 than those who were not. Marital status did not have any effect on PCS-12. The number of chronic medical conditions was negatively associated with both MCS-12 and PCS-12. Results also showed an association between general satisfaction with care and MCS-12 but not PCS-12. Patients born in Australia were likely to have higher MCS-12 than those born in non-English-speaking countries but country of birth was not associated with PCS-12.

Patients from smaller general practices (1–3 GPs) reported lower PCS-12 and MCS-12 compared with those from larger practices. Practice location had no relationship with either PCS-12 or MCS-12.

### Variance components

Ninety seven per cent of the total variance in PCS-12 was at the patient level, the remaining 3% variance (Intra-cluster correlation (ICC) = 0.03) was at the practice level. For MCS-12, the corresponding figures were 99% at patient level and 1% (ICC = 0.01) at practice level (baseline model in Table 4). At the patient level (level 1) 42% and 21% of the variance respectively among patients for PCS-12 and MCS-12 were explained by the independent variables used in the analysis (Table 4). At the practice level (level 2), 73% and 49% of the variance among practices for PCS-12 and MCS-12 were explained by the variables used in the analysis (Table 4).

## Discussion

The SF-12 is a subjective measure of health that can be influenced by a respondent's perceptions, expectations and interpretations about health [12]. Nonetheless, the scale has become one of the most widely used HRQOL measures. This study provides the first comprehensive data on physical and mental health of chronically-ill patients in Australia.

While 103 Divisions of General Practice were approached to participate in recruiting practices to the study, only 27 Divisions agreed to participate and there were no remote area practices in the sample. Practices that volunteered to participate may not be representative of all practices within Australia or within the participating Divisions. However, the proportion of practices that were solo, or large (4 or more GPs) was similar to that reported in other studies [33]. Patients that the practice identified as being unable to read English were excluded from the study. Although the response rate of 61% was comparable with other studies [30], it is possible that some of those not responding may have had different views of their physical and mental health from those who responded. For example, 20% of non-respondents were younger than 40 years compared with 10% of respondents. These younger non-respondents would have primarily have had a diagnosis of asthma. We adjusted for these differences in distribution between the total sample (14% from 18–39 age group) and respondents by giving greater weight to younger respondents. The results showed the mean difference between unadjusted and adjusted was 0.51 for PCS-12 (P < 0.05) and 0.31 for MCS-12 (P > 0.05). Finally there may have been other practice and patient factors important to patient HRQOL assessments such as patient co-payments and availability which were not specifically measured in this study and warrant further exploration in the Australian context.

It is recommended that standard US-derived scoring of the SF-12 summary scores (scores with a mean of 50 and standard deviation of 10 in the U.S. general population) can be compared and interpreted across countries [19]. For example, the average unadjusted PCS-12 (42.7) and MCS-12 (50.2) for type 2 diabetes patients were consistent with those of Canadian (PCS_12 = 42.0 and MCS-12 = 48.5) type 2 diabetes patients (Table 1) [34]. The overall mean of PCS-12 (42.4, SD = 11.8) and MCS-12 (49.1, SD = 11.1) of chronically-ill patients in the study were less than those of U.S. general population (mean = 50, SD = 10). The difference for PCS-12 was clinically significant with effect size of 0.69 and that for MCS-12 was not clinically significant (effect size = 0.09). The effect size of more than 0.5 is considered to be clinically significant [8]. The difference between the PCS-12 in this study and that of the Australian general population (PCS-12 = 50.9, SD = 8.7) [35] was clinically significant (effect size = 0.82). However, the difference between the MCS-12 and that of the general population (MCS-12 = 50.3, SD = 9.9) was clinically not significant (effect size = 0.11) [35].

We also examined ceiling (highest possible score) or floor effects (lowest possible score) of 12 items and their loadings on each factor. All floor effects were < 15% except for two PF items with the limited answering options (both items are on a 3-point scale) but ceiling effects for some items (item/items of PF, RP, BP, RE and SF) were >15%. Such ceiling effects are seen in both the SF-36 [36] and SF-12 [37]. Large ceiling effects are undesirable because they reduce scale sensitivity [36]. Ceiling or floor effects were less than 0.04% for both PCS-12 and MCS-12. VT and SF were the most confounded in PCS-12 and MCS-12 (Table 2). Principal component scores offer a solution to this confounding.

The practice level variance for PCS-12 was small but significant even after adjustment for patient and practice characteristics which supports the choice of multilevel analysis. That of MCS-12 was not significant after adjustment. The large patient level variance is consistent with other studies [14,30]. This suggests that most of the differences between patients may be related to patient selection rather than differences in the care provided by practices. There was a negative effect of size of practice on both PCS-12 and MCS-12 that may reflect the decreased continuity of care provided in larger practices and patients with poor health may have self-selected smaller practices for better continuity of care [38]. Most of the variance in both PCS-12 and MCS-12 was related to patient level factors such as age, socio-economic status and ethnicity. Socio-economic status was measured by employment, home ownership and education. The effects of home ownership and education were clinically not significant (effect size < 0.5), but the effect of employment was clinically significant for PCS-12. Further studies of this type are required to identify other variables that explain the variance in MCS-12. By contrast practice size and other independent variables explained most of the practice level variance in both PCS-12 and MCS-12.

Our finding that PCS-12 was lower in older age while MCS-12 was higher in older age groups is consistent with previous research [7,9,11,14,18,39]. In clinical practice this underlines the importance of looking for psychological distress in association with chronic illnesses such as hypertension/ischaemic heart disease, diabetes and asthma, and in younger age groups and unemployed.

Consistent with other research, lower socio-economic groups reported lower PCS-12 and MCS-12 [7,11,14]. Our previous research indicated that while Australian general practitioners working in low income areas provided fewer long consultations, other markers of process of care for diabetes were better [40,41]. Thus while the socio-economic gap in HRQOL may not be attributable to differences in quality of general practice care, it does suggest that greater effort is needed to improve outcomes for low socio-economic chronically-ill patients and that GPs working with these patients may require additional support such as practice nurses or allied health providers.

People from non-English-speaking backgrounds had lower MCS-12 but not PCS-12. Again this is consistent with other research [7,9]. We do not have information on the circumstances of patient migration (especially the proportion who were refugees), however it is possible that the worse mental health may have been due to acculturation issues. Patients from non-English-speaking backgrounds were also less satisfied with their care [38].

Some studies have shown a significant interaction effect between gender and employment indicating employed men enjoy higher levels of general well-being [15,28]. In this study there was an interaction between gender and employment status with the negative impact of unemployment being greater in male than female patients. Male employed respondents were likely to have higher physical and mental health than unemployed males (large effect sizes of 1.37 and 0.70 for PCS-12 and MCS-12 respectively). The effect of employment was less on females. This may be because the significance of work and its impact on household income may be greater in chronically-ill older men than in women [27]. The 'unemployed' category in our study included people who were unable to work due to sickness or disability (11% of males and 7% of females) and looking after family or home (1% of males and 19% of females). Probably, this might explain some of the interaction.

### Policy and practice implications

Based on the results of the analysis reported here, the SF-12 and its component scales appear to be valid and useful tools to use in identifying differences in quality of life of the chronically-ill Australian population on the basis of social determinants of health [7]. Known group comparisons based upon differences in general health, age, socio-economic status, and number of medical conditions yielded support for the construct validity of the SF-12 in this data [8,10,42]. Further, our data showed an association between general satisfaction with care or marital status with mental health but not with physical health confirming the results of previous studies [43-45]. In our sample it appeared that the dimensions were discriminative enough to distinguish between respondents with a single illness and with two or more illnesses or low and high socio-economic status or younger and older respondents. Further, there was strong association between SF-12 summary scores and self-rated general health status collected independently using GPAS for the same respondents (clinically significant large effect sizes of 1.27 and 0.79 for PCS-12 and MCS-12 respectively). This ability to discriminate between groups means that clinicians can use scores better to understand the functional status and health care needs of at-risk subgroups, and also enables policy makers to measure clinical effectiveness [10]. The ability to detect previously hypothesized differences or associations between variables showed the construct validity of SF-12 in Australia [6]. Further, the results suggest that the SF-12 has construct validity when applied to an Australian primary care population with chronic illness.

## Conclusion

The strong association between patient characteristics such as socio-economic status, age, and ethnicity and SF-12 physical and mental component summary scores underlines the importance of considering these factors in the management of chronically-ill patients in general practice and adjusting for them in the assessment of the performance of practices. The SF-12 appears to be a valid measure for assessing HRQOL of Australian chronically-ill patients.

## List of abbreviations

BP: Bodily Pain; GH: General Health; GP: General Practitioner; GPAS: General Practice Assessment Survey; HRQOL: Health-Related Quality of Life; ICC: Intra-Cluster Correlation; MCS: Mental Health Component Summary; MCS-12: Mental Component Score derived from the SF-12; MH: Mental Health; PCS: Physical Component Summary; PCS-12: Physical Component Score derived from the SF-12; PCS-36: Physical Component Score derived from the SF-36; PF: Physical Functioning; RE: Role Emotional; RP: Role Physical; RRMA: Rural, Remote and Metropolitan Area; SD: Standard Deviation; SF: Social Functioning; SF-12: Short Form 12-item Health Survey; SF-36: Short Form 36-item Health Survey; VT: Vitality.

## Competing interests

The authors declare that they have no competing interests.

## Authors' contributions

UJ contributed to data analysis, interpreting the data and drafting the manuscript. UJ and MH made substantial contributions to conception and design of the study. JP, CA, CH were involved in the data collection. All authors were involved in drafting the manuscript or revising it critically for important intellectual content. All authors have read and approved the final version of the manuscript.
